# Analysis of Colorectal Carcinogenesis Paradigm between Cold Constitution and Heat Constitution: Earlier ECM Collagen Deposition

**DOI:** 10.1155/2021/5547578

**Published:** 2021-07-17

**Authors:** Feifei Nong, Yuqi Liang, Shangping Xing, Huixuan Li, Xizheng Lin, Jingchun Qin, Fengliang Hu, Bin Wen

**Affiliations:** ^1^Pi‐Wei Institute, Science and Technology Innovation Center, Guangzhou University of Chinese Medicine, Guangzhou 510006, China; ^2^The Second Clinical College of Guangzhou University of Chinese Medicine, Guangzhou 510282, China; ^3^Research Center of Chinese Herbal Resource Science and Engineering, Key Laboratory of Chinese Medicinal Resource from Lingnan, Ministry of Education, Guangzhou University of Chinese Medicine, Guangzhou 510006, China; ^4^The First Affiliated Hospital of Guangzhou University of Chinese Medicine, Guangzhou 510006, China

## Abstract

Colorectal cancer (CRC) is a common malignant tumor around the world. Studying the unique constitution of CRC patients is conducive to the application of personalized medical treatment for CRC. The most common types of constitution in CRC are cold and heat constitution. A previous study has suggested that the malignant progression in cold and heat constitution CRC are different; however, the mechanism remains unclear. The tumor microenvironment (TME) is likely to vary with each individual constitution, which may affect the tumor growth in different constitutions. The extracellular matrix (ECM), the most important component of TME, plays a critical role in disease progression and outcome in patients with CRC. Moreover, collagen, the major component of the ECM, determines the main functional characteristics of ECM and tissue fibrosis caused by collagen deposition, which is one of the signs of CRC malignant progression. This study aimed to explore the mechanisms leading to different colorectal carcinogenesis paradigms between the cold constitution and heat constitution within the context of ECM collagen deposition. We established the CRC rat models and enrolled 30 CRC patients with cold and heat constitution. The collagen-related parameters were detected by using Sirius red staining combined with polarized light microscope, and expressions of collagen (COL I and COL III) and lysyl oxidase (LOX and LOXL2) were determined using immunohistochemistry, while the mRNA levels of COL1A1, COL3A1, LOX, and LOXL2 were measured by qRT-PCR. We found that a higher degree of collagen deposition in the cold-constitution group. The results suggest cold and heat constitution may affect the colorectal carcinogenesis paradigm by influencing the early collagen deposition in colon tissue. The study may provide an effective idea for clinicians to improve the prognosis of CRC patients with different constitutions.

## 1. Introduction

Colorectal cancer (CRC) is a common malignant tumor around the world, and its incidence ranks third among malignant tumors, with a 5-year survival rate of metastatic patients at less than 10% [1]. Previous studies indicated that CRC in only a minority of patients is caused by the accumulation of genetic, epigenetic alterations, whereas the majority are linked to environmental factors such as dietary intake [2–4]. Diet habit may affect the constitution, and constitution refers to the relatively stable inherent characteristics in terms of morphological structure, physiological function, and mental state that differ individually in humans. Body constitution lays the foundation for diagnosis, treatment, and disease prevention, and different types of constitutions lead to differences in the individuals' susceptibility to disease [5, 6]. Moreover, examining CRC patients' unique constitution can promote effective health management and benefit the application of personalized medicine in the treatment of CRC significantly [7].

A study on constitutional classification has indicated that each classification includes face, appearance, spirit, color, tongue, pulse, and urinary and fecal discharges of an individual [6]. For constitution identification, the most important and unique constitution is the cold and heat constitution. Increasing evidence has pointed out that long-term spicy food intake easily induces heat constitution, whereas the long-term cold drink intake easily induces cold constitution [8–10]. Cold-constitution patients are more likely to have an intolerance to cold, paler complexion, loose or watery stool, clear urine, paler tongue, white tongue coating, slow pulse, cold limbs and so on. Heat-constitution patients are more likely to have a tolerance to cold or intolerance to heat, dry mouth and thirst, redder complexion, constipation or dry stool, yellow urine, impatience, redder tongue and so on. Moreover, the cold constitution is related to low and slow metabolism, whereas the heat constitution is related to an increase in the metabolic rate [11, 12]. A previous study has suggested that the malignant progression in cold- and heat-constitution CRC is different [13]; however, the mechanism is still unclear.

The tumor microenvironment (TME) is likely to vary with each individual constitution, which may affect the tumor growth depending on different constitutions [8]. Extracellular matrix (ECM), as the main component of TME, is a dynamic compartment that regulates cell functions such as proliferation, adhesion, differentiation, migration, and proliferation, affecting the physiological and pathological states of the tissue [14, 15]. ECM homeostasis is essential for organ development and function under physiological conditions, while its sustained modification or dysregulation can result in pathological conditions. Tumor stroma and biomechanical abnormalities developed during tumor growth comprise dominant regulators of cancer progression; ECM is no longer an inert scaffold but plays an active role in the control of tumor cells and tumor growth [16–18]. Collagens, as the major constituents of the ECM, represent as much as 30% of total mammalian protein mass and dictate the primary functional properties of the matrix [19]. Collagen remodeling can create space for cells to migrate, produce substrate cleavage fragments with independent biological activity, modify adhesion to regulate tissue architecture, and activate, deactivate, or alter the activity of signaling molecules [20, 21]. Indeed, changes in the deposition or degradation of collagen can lead to the loss of ECM homeostasis. Tissue fibrosis and stiffness caused by collagen deposition and crosslinking is one of the signs of CRC malignant progression [22, 23].

In our previous study [24], ice water and capsaicin were used to induce rat models with basic symptoms of cold- and heat-constitution in order to simulate the cold constitution and heat constitution induced by the long-term cold drink and long-term spicy food intake, respectively, in human beings. Moreover, we found that the length of the colon was shorter in the cold-constitution CRC model than that in the heat-constitution model, which suggested more severe fibrosis. Therefore, we aimed to explore the mechanisms leading to the different colorectal carcinogenesis paradigm between the cold constitution and heat constitution within the context of ECM collagen deposition.

## 2. Materials and Methods

### 2.1. Experiment Reagent

Capsaicin (97% purity, C10209596) was purchased from Shanghai Macklin Biochemical Technology Co., Ltd. Ethanol (P201450) was purchased from Tianjin Fuyu Chemical Co., Ltd. Commercial refrigerator display cabinet was purchased from Beijing Snowflake Electric Appliance Group Co., Ltd. DMH (D161802, Sigma, USA) and immunohistochemical kit (SP9000) were purchased from Zhongshan Jinqiao Co., Ltd., China. Ultra-micro Na^+^K^+^-ATP, Ca^2+^Mg^2+^-ATP, total ATP enzyme assay kit (A070-6-2), LDH enzyme assay kit (A020-2-2), and SDH enzyme assay kit (A022-1-1) were purchased from Nanjing Jiancheng Institute of Biological Engineering, China. Electronic anal thermometer was purchased from Shangnong Electronic Technology Co., Ltd. (SNT, China).

### 2.2. Patients and Clinical Data

A total of 30 patients (age, 26–86 years) at the First Affiliated Hospital of Guangzhou University of Chinese Medicine (Guangzhou, China) between July 2019 and February 2020 were enrolled in the present study. The participant inclusion and exclusion criteria are listed in Table 1. All patients were pathologically diagnosed with CRC. Patients who underwent neoadjuvant chemotherapy or radiotherapy or had a previous history of malignancy were excluded. Clinical and pathological data, including pathology reports, sex, age, macroscopic classification, tumor location, tumor size, tumor differentiation, lymphatic infiltration, and depth of invasion, were collected from medical records. The American Joint Commission on Cancer TNM staging system was used to clinically stage the tumor [25]. This study was approved by the Ethics Committee of First Affiliated Hospital of Guangzhou University of Chinese Medicine (No. Y [2019]172) and written informed consent was obtained from all patients. The study was performed in accordance with the Declaration of Helsinki.

### 2.3. Distinguishing between Cold Constitution and Heat Constitution in CRC Patients

Distinguishing between cold-constitution and heat-constitution patients was completed by two experienced physicians according to the patients' daily habits (habitually drank cold water or ate spicy food) and clinical signs. Vital signs of cold constitution were as follows: habitually drinking cold water 4–6 cups (about 800–1200 mL) per day or eating cold food (e.g., ice-cream), intolerance to cold, paler complexion, loose or watery stool, and clear urine. Main symptoms of heat constitution were as follows: habitually eating spicy food at least 5 days per week, tolerance to cold or intolerance to heat, dry mouth and thirst, redder complexion, dry and hard stool, and deep yellow urine. The people who both drank cold water and ate spicy food were excluded. If the constitution diagnoses of these two experts were different, then a third expert was invited to help classify a patient as cold or heat. All patients included in the study had no declared history of smoking or drinking alcohol and were categorized as normal weight (BMI = 18.5–24.9).

### 2.4. Experimental Animals and Treatments

#### 2.4.1. Experimental Animals

Ninety-six male Wistar rats, 7-week-old weighing 200 ± 20 g, were obtained from the Experimental Animal Center of Southern Medical University [certificate of quality: SCXK (Yue) 2016–0041]. All rats were housed in cages (25 × 30 × 30 cm) under pathogen-free conditions at 25°C, 40–60% relative humidity, and 12-hour light/dark cycle in experimental Animal Center of Guangzhou University of Chinese Medicine. All rats had ad libitum access to standard rodent chow and filtered water and were acclimatized for 1 week prior to the initiation of the experiment. The use of laboratory animals was checked by the “Institutional Animal Ethical Committee (IAEC)” and all procedures were approved by the Ethics Committee of Guangzhou University of Chinese Medicine and performed according to the “Principles of Laboratory Animal Care” and specific national laws where applicable. All experimental protocols and handling of the animals were following the Guide for the Care and Use of Laboratory Animals [26].

#### 2.4.2. Preparation of Carcinogen

DMH was weighed and dissolved in normal saline to ensure the stability of the chemical before use with a final concentration of 2%. Moreover, the pH was adjusted to 6.5 each time with 1 M NaOH solution.

#### 2.4.3. Experimental Procedure and Groups

Ninety-six rats were randomly divided into four groups: control group, model group, heat-constitution model group, and cold-constitution model group with 24 rats each. Random numbers were generated using the standard = RAND () function in Microsoft Excel.

Control group: rats received a normal diet along with distilled water (10 mL/kg b.wt.).

Model group: rats were administered with subcutaneous injection of DMH at a dose of 30 or 35 or 25 or 20 mg/kg b.wt. Once a week for 12 weeks from 6th week on.

Heat-constitution model group (DMH + capsaicin): rats were administered with DMH as in the model group and also fed 20% ethanol (10 mL/kg b.wt.) every day for the first week, and then 0.9 mg/mL capsaicin, dissolute in 30% ethanol (9 mg/kg b.wt. orally), was intragastrically administered to rats every day from the second week on till the end of the experiment, which is called the heat M group.

Cold-constitution model group (DMH + ice water): rats were administered with DMH as in the model group and also fed 0°C ice water (10 mL/kg b.wt.) five times every day till the end of the experiment, which is called cold M group.

**Figure 1:**
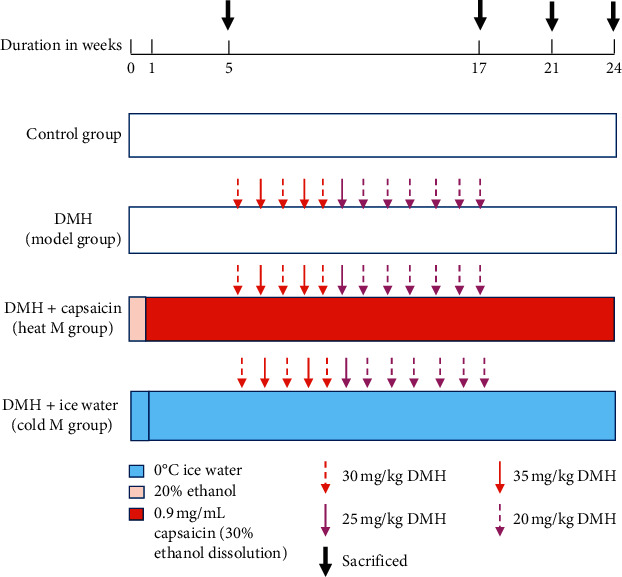
The schematic representation of the experimental design.

The details of the specific modeling method of each group are shown in Figure 1. Body surface symptoms and anal temperature of rats were recorded, and the basic symptoms scores were counted to evaluate the cold constitution or heat constitution. The rats were anesthetized with mild anesthesia and sacrificed by cervical dislocation at the end of 5th, 17th, 21st, and 24th week, respectively.

### 2.5. Activity of Energy Enzyme

The contents of Na^+^K^+^-ATPase, Ca^2+^Mg^2+^-ATPase, total ATPase, LDH enzyme, and SDH enzyme in the supernatant of colon homogenate were determined by the protocol.

### 2.6. Morphological and Histological Evaluation

For morphological evaluation, rat colons were longitudinally dissected from the anal to the cecum and then washed with PBS. The colons were spread out on cleaning tissue paper. The length of colons was recorded, and the number of colon tumors was recorded for tumor incidence and the size of the tumors was measured using a caliper paper.

For histological evaluation, H&E staining was done to observe the colonic histology differences by the method of Martin B [27]. Moreover, the H&E results were observed under a 40x magnification light microscope (Olympus, BX51) to investigate the pathologic structure of the colonic mucosa of rats. Moreover, the determination of aberrant crypt foci (ACF) was performed according to the method described by Bird and Lafave [28]. The formalin-fixed colon was then stained with 0.2% methylene blue for 3 min and then viewed under a microscope (Olympus, BX51). The diagnosis of colonic pathology and ACF was performed by two pathologists. Moreover, the number of ACFs was counted as described by Sivaranjani et al. [29].

### 2.7. Sirius Red Staining

Sirius red staining method was applied to detect the collagen morphology and related parameters of colon tissues. After the colon specimens were made into paraffin sections, they were immersed in the Sirius red dye solution for 8 min. The stained sections were observed using an ordinary light microscope (Olympus, BX51) and a polarized light microscope (Olympus, IX73) [30]. Collagen-related parameters include density, width, length, stiffness, and angle [31]. The density of collagen fibers in the tumor stroma was determined by ImageJ software, while the other four parameters were detected by CT-Fire software.

### 2.8. Quantitative Real-Time PCR Analysis

Total RNA from tissues was extracted using the RNAEX reagent (Accurate Biotechnology, China) according to the manufacturer's instructions. Moreover, they were reverse-transcribed into cDNA using the Evo M-MLV RT Premix (Accurate Biotechnology, China) and a RT-PCR system (TaKaRa, Japan) in accordance with the manufacturer's recommended protocols. The PCR reaction was using SYBR Green Premix Pro Taq HS qPCR Kit (Accurate Biotechnology, China) in a 20 *μ*L volume of the PCR reaction solution. Quantitative PCR was performed using a RT-PCR system (BioRad, Singapore). The amplification conditions after an initial denaturation step for 90 s at 95°C were 40 cycles of 10 s at 95°C for denaturation and 34 s at 60°C for elongation. GAPDH was used as the reference gene for the calculations. The results were expressed relative to GAPDH with the comparative CT method. The primer sequences are listed as follows: for rat, GAPDH: 5'-TGCCCTCATGTTCCTGATAAAT-3' and 5'-CATTACATCACAGCTTTCCAGG-3', COLI: 5'-AGGCATAAAGGGTCATCGTGGCTT-3' and 5'-AGTCCATCTTTGCCAGGAGAACCA-3', COL III: 5'-GGTTTGGAGAATCTATGAATGGTGG-3' and 5'-GCTGGAAAGAAGTCTGAGGAAGG-3', LOX: 5'-TGTTGAGGCAAATACAAACCC-3' and 5'-ATTCGCTACACAGGACATCA-3', LOXL2: 5'-AGCCTATAAGCCGGAGCAAC-3' and 5'-GTCCCACTTGTCATCGCAGA-3'; for human, GAPDH: 5'-GGAGCGAGATCCCTCCAAAAT-3' and 5'-GGCTGTTGTCATACTTCTCATGG-3', COLI: 5'-GAGGGCAACAGCAGGTTCACTTA-3' and 5'-TCAGCACCACCGATGTCCA-3', COL III: 5'-CCACGGAAACACTGGTGGAC-3' and 5'-GCCAGCTGCACATCAAGGAC-3', LOX: 5'-TTCTTACCCAGCCGACCAAGATA-3' and 5'-GTGTTGGCATCAAGCAGGTCA-3', LOXL2: 5'-GTGGATCTGGCACGACTGTCA-3' and 5'-TTGAGGTTCAGCAGGTCATAGTGG-3'.

### 2.9. Immunohistochemical Staining

Immunohistochemical staining in the study was done by the method of Mansour et al. [32]. The slides were observed under the light microscope (Olympus). Primary antibodies of COL I (dilution 1 : 500, Abcam, ab34710); COL III (dilution 1 : 100, Abcam, ab7778), LOX (dilution 1 : 50, Abcam, ab31238), and LOXL2 (dilution 1 : 500, Abcam, ab197779). All other chemicals and reagents used in immunohistochemical staining experiments are of the highest purity grades available on the market. Moreover, the results were observed under a 40x magnification light microscope (Olympus, BX51) to investigate the expressions in rats and 20x magnification in human tissues. For immunohistochemical quantification, images of three randomly selected microscopic fields per slide were evaluated by independent pathologists. Image Pro Plus 6.0 (Media Cybernetics, Inc.) was used for digital image analysis. The scores for staining intensity and the percentage of positive cells were multiplied; the yellow density reflects the expression level of the target protein. The expression levels of COL I, COL III, LOX, and LOXL2 were quantified via the average optical density (AOD).

## 3. Statistical Analysis

Data were described using mean (standard deviation), median (range), or frequency (percentage). The difference between four groups (control group, model group, cold M group, and heat M group) was evaluated by one-way analysis of variance (ANOVA, normal distribution) or Kruskal–Wallis (nonnormal distribution). Tukey's test (equal variances assumed), Dunnett's T3 test (equal variances not assumed), or Dunn-Bonferroni post hoc comparison (nonnormal distribution) was used for post hoc comparison. The comparison between two groups (cancer and normal) was analyzed by parametric paired *t*-test or nonparametric Wilcoxon's test. The independent-samples *t-*test and the Mann–Whitney *U* test were applied for the comparison between two groups (cold M group and heat M group). The associations between the clinical parameters and immunohistochemical results were analyzed using the *χ*2 test. Statistical analyses were conducted using SPSS version 25.0 for Windows (SPSS, Inc.). Statistical analysis was performed: ^*∗∗*^*P* < 0.01; ^*∗*^*P* < 0.05; ^*ns*^*P* > 0.05.

## 4. Results

### 4.1. Basic Symptoms and Temperature of Rats

**Figure 2:**
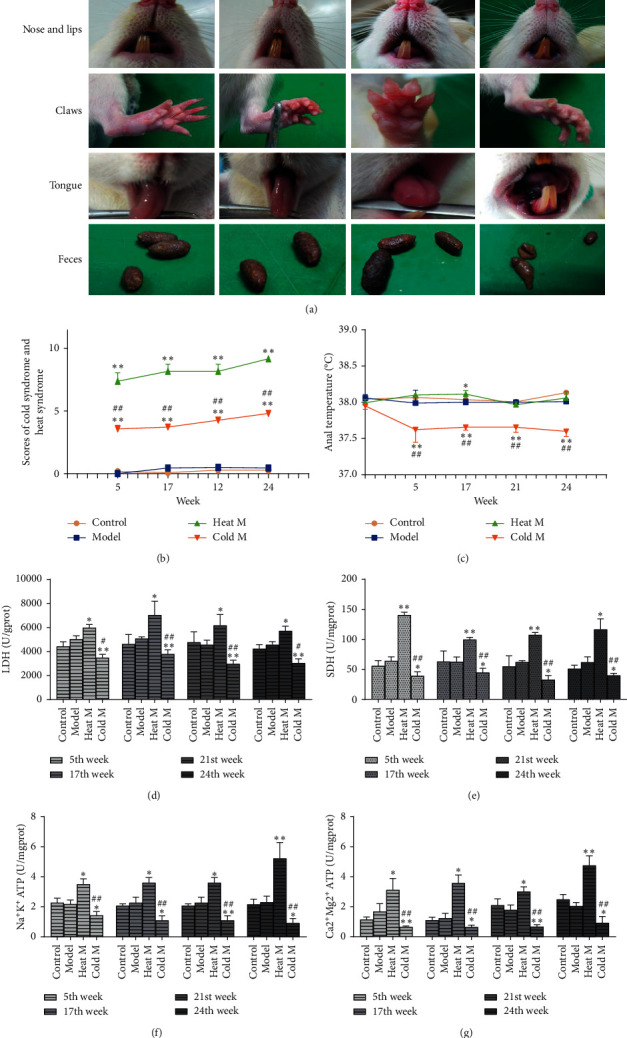
The changes of body surface symptoms and physiological and biochemical indexes of different groups. (a). The body surface symptoms of control group, model group, heat M group, and cold M group at the 24th week. (b). The scores of cold constitution and heat constitution of rats. (c). The changes of anal temperature of rats in each group. (d). The LDH activity of rats in each group. (e). The SDH activity of rats in each group. (f). The Na^+^K^+^ ATP activity of rats in each group. (g). The Ca^2+^Mg^2+^ ATP activity of rats in each group. Compared with the model group: ^*∗∗*^*P* < 0.01, ^*∗*^*P* < 0.05; compared with the heat M group: ^##^*P* < 0.01, ^#^*P* < 0.05.

As shown in Figure 2(a), from the 5th week on, the cold-constitution rats were more likely to have indifference, soft rotten stool, clear urine, green and purple tongue, lips, nose and toe claws turn pale, cold limbs, or other symptoms until the end of the experiment. The heat-constitution rats were more likely to have redder complexion, dry and hard stool, dark urine, irritable, redder tongue, lips, nose and toe claws turned red, and tongue turned red and swollen, which is similar to the cold and heat constitution described in previous research [11]. Moreover, the control and model groups showed mild temper, normal feces, pink tongue, pink lips and nose, and pink toe claws.

According to the standards of basic symptoms scores of cold constitution and heat constitution [24, 33] (Table 2), when the score of the animal model is higher than 6, the rats will be evaluated as the heat-constitution ones; when the score is more than 3 and less than 6, then they will be classified as the cold-constitution ones. The scores of physical signs were shown in Figure 2(b). The scores of the control and model groups were less than 1. The basic symptoms scores of cold-constitution and heat-constitution groups were stable until the end of the experiment. Compared with the model group, the score of the cold model group was between 3 and 6, and the score of the heat model group was higher than 6, which was significantly higher than that that in the cold M group and the model group (^*∗*^*P* < 0.05, ^*∗∗*^*P* < 0.01, vs. model group; ^#^*P* < 0.05, ^##^*P* < 0.01, vs. heat M group). The anal temperature of rats was regularly measured and recorded (Figure 2(c)). We found that during the whole experiment, the anal temperature of rats in the control group and the model group maintained at about 38.0°C, and there was no statistical difference between the two groups. Compared with the model group, the heat M group showed a little higher with a significant difference at the 17th week. The anal temperature of the cold M group was relatively low and remained about 37.6°C from the 5th week to the end of the experiment compared with the model group and the heat M group showing statistical difference (*P* < 0.01), which is consistent with the description of the temperature characteristics of cold constitution and heat constitution in a previous study [11]. The results of basic symptoms scores showed that the rat models with cold constitution and heat constitution were established successfully.

### 4.2. The Activities of Energy Enzymes in Rats

Lactate dehydrogenase (LDH) is a glycolytic enzyme, which is an important determinant of whether consumed glucose is converted into energy by aerobic or anaerobic glycolysis [34]. The activity of succinic dehydrogenase (SDH) directly affects the process of oxidative phosphorylation of mitochondria, and the increase of SDH activity means the enhancement of cell energy metabolism [35]. ATP is the main medium of energy conversion and metabolism in mammals, which is the catalyst of energy production [36]. Na^+^-K^+^-ATP and Ca^2+^-Mg^2+^-ATP provide conditions for cells to maintain normal morphology and function. It is reported that the activity changes of ATP, LDH, and SDH can be used as evaluation indexes of cold-constitution and heat-constitution models [37, 38]. The contents of Na^+^-K^+^-ATP, Ca^2+^-Mg^2+^-ATP, SDH, and LDH of the colon tissues in each group were shown in Figures 2(d)–2(g). Compared with the control group, the activities of Na^+^-K^+^-ATP, Ca^2+^-Mg^2+^-ATP, SDH, and LDH in the model group had no significant difference; however, the activities of Na^+^-K^+^-ATP, Ca^2+^-Mg^2+^-ATP, SDH, and LDH decreased significantly in the cold M group than in the model group. There was no significant difference in the activity of Na^+^-K^+^-ATP in the heat M group, but the activities of Ca^2+^-Mg^2+^-ATP, SDH, and LDH increased significantly (*P* < 0.05) than those in the model group. Compared with the cold M group, the activities of Na^+^-K^+^-ATP, Ca^2+^-Mg^2+^-ATP, SDH, and LDH in the heat M group were significantly higher than those in the cold M group. Our results showed that the energy metabolism of the cold M group slowed down and inhibited the activity of the energy enzyme, while the energy metabolism of the heat M group was faster than the normal level. Kim et al. have indicated that cold constitution is related to low and slow metabolism, whereas the heat constitution is related to an increase in the metabolic rate [11]. Our results are basically consistent with the description of heat constitution and cold constitution in the previous study.

### 4.3. Colonic Morphology Analysis in Rats

**Figure 3:**
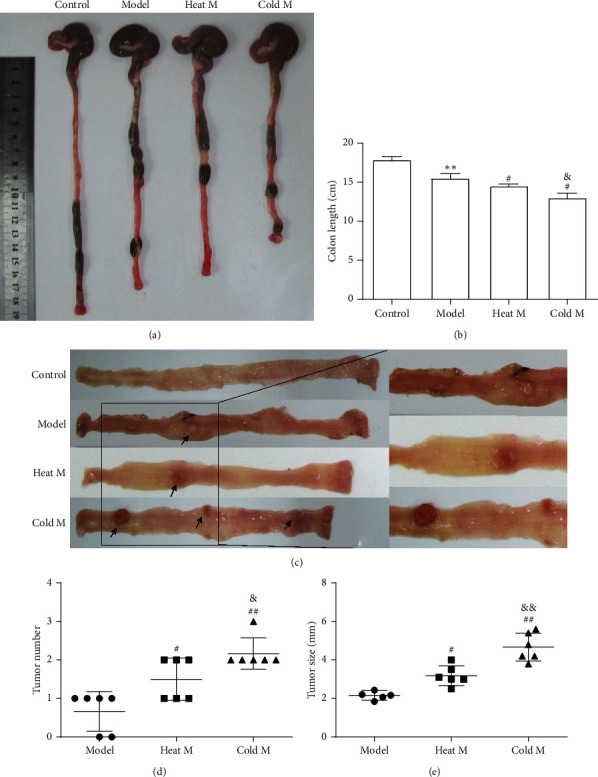
Colonic morphology and tumor burden in different groups at the 24th week. (a). The appearance of colonic tissue in each group were compared at the 24th week. (b). The lengths of colonic tissue in each group were compared at the 24th week. (c). Morphological view of colonic tumors. (d). Average tumor number and tumor size (e) in each group. Compared with the control group: ^*∗∗*^*P* < 0.01, ^*∗*^*P* < 0.05; compared with the model group: ^#^*P* < 0.05; compared with the heat M group: ^&^*P* < 0.05.

The colonic morphology and the colonic length of rats in each group were observed and measured at the 24th week of the experiment, respectively (Figure 3(a) and 3(b)). Compared with the control group, the colonic length in the model group was significantly shorter (*P* < 0.05). Moreover, the colonic length of rats in the heat M group and the cold M group was shorter and statistically different compared with that in the model group (*P* < 0.05). Colon length, a marker of intestinal fibrosis, was significantly shorter in the cold M group than any other groups, which indicated that the degree of intestinal fibrosis was the highest in the cold M group.

In addition, no visible colon tumor was found in the control group during the experiment. As shown in Figures 3(c) and 3(d), at 24th week, the average tumor number was higher among cold M (2.6 ± 0.41) and heat M (1.5 ± 0.44) group as compared to the model group (0.67 ± 0.34), and that in the cold M group was higher than that in the heat M group. On the other hand, the average tumor size of the model group was 2.16 ± 0.25 mm and that of the cold M and heat M groups was 4.76 ± 0.76 mm and 3.22 ± 0.56 mm, respectively. These data suggest that there was a bigger tumor size in rats of the cold M and heat M groups than in the model group. Moreover, there was a higher tumor incidence of the cold M and heat M groups than that in the model group, and there was no difference in tumor incidence between cold M and heat M groups (Table 3).

### 4.4. Histopathological Analysis in Rats

**Figure 4:**
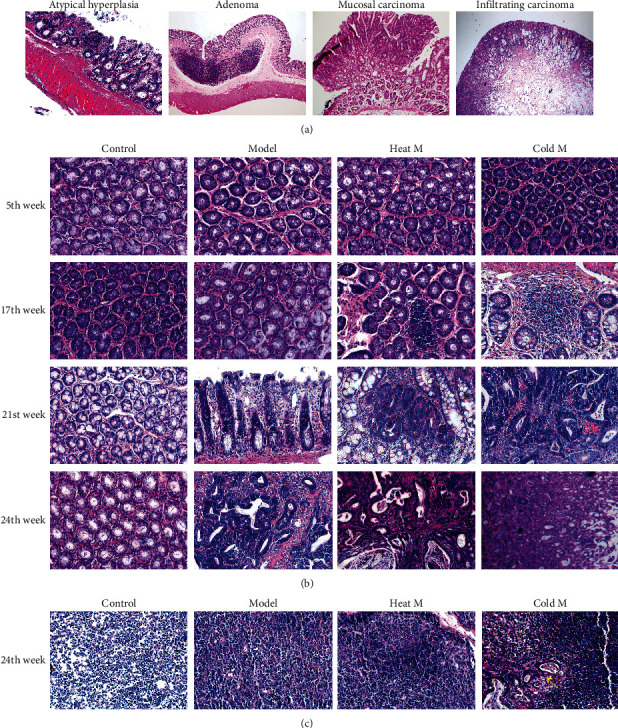
The H&E images of different groups. (a). Representative sections showing the histopathology of the colonic neoplastic lesions (×200). (b). Colon pathology of different groups (×400). (c). The pathology of Colonic lymph node in different groups at the 24th week (×400).

Representative sections showing the histopathology of the colonic neoplastic lesions are shown in Figure 4(a). The pathology of rat colons at the 5th, 17th, 21st, and 24th weeks was examined with H&E staining shown in Figure 4(b). According to the criteria of histological diagnosis of tumor [39], the pathological classification of each group can be seen in Table 4. All the rats in the control group had no adenoma or carcinoma during the whole experiment. Moreover, there were no obvious abnormalities in colonic pathology in each group at the 5th week under the light microscope. After the 12th injection of DMH, at the 17th week, half of the rats in the model group showed atypical hyperplasia. Moreover, the rate of atypical hyperplasia in heat M and cold M group was 100%, showing an increase of crypt cells and layers and irregular shape. At 21st week, 66.7% of the rats in the model group had many heteroepithelial cells in the colonic epithelium and one or more large nucleoli in the pleomorphic cystic nucleus, demonstrating carcinoma adenoma in situ. Half of the rats in the heat M group had mucosal carcinoma, featuring less mucus secretion of colonic epithelial cells, compact and irregular arrangement of glandular ducts, and obvious invasion of cancer cells into lamina propria. Half of the rats in the cold M group had invasive mucosal infiltrating carcinoma, presenting with abnormal tubular or mucinous structures invading through the muscularis mucosa or deep intestinal wall. At the end of the experiment, at 24th week, the colon of 66.7% rats in the model group showed mucosal carcinoma. The infiltrating carcinoma rate in the mucosa of rats in the heat M group was 66.67%, and the other 33.33% of rats showed mucosal carcinoma. In the cold M group, 83.33% of the rats were considered to be mucosal infiltrating carcinoma. The mesenteric lymph nodes stained with H&E were observed under a light microscope (Figure 4(c)). We found no lymphatic metastasis in the model group and heat M group, while cancer cell infiltration was found in the lymph node of the cold M group at the 24th week and the lymph node metastasis rate was 33.33% (Table 4). These results showed that the different pathologic characteristics of CRC were shown between the heat M and cold M groups. The cold M group showed a higher malignant degree of CRC than any other group, followed by the heat M group.

### 4.5. The Screening of Early Aberrant Crypt Foci in Rats

**Figure 5:**
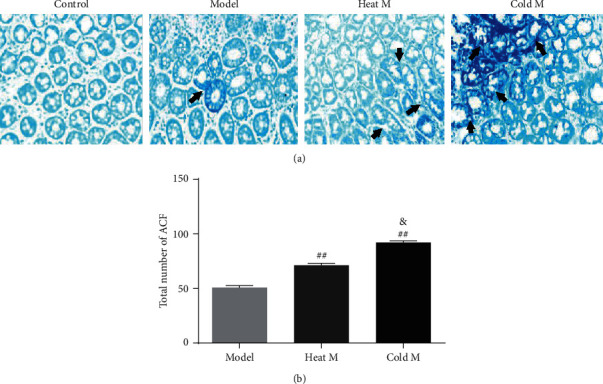
The determination of ACF in different groups. (a). Methylene blue staining for ACF in the sectioned rat colon (×400): control rat showed normal crypts; model rat, heat M rat, and cold M rat showed ACF (arrow) at the 17th week. (b). Bar graph of ACF with different crypt multiplicity. Compared with the model group: ^##^*P* < 0.01, ^#^*P* < 0.05; compared with the heat M group: ^&&^*P* < 0.01, ^&^*P* < 0.05.

Figure 5 summarizes the data regarding ACF at 17th week. The total number of ACFs detected in the model group was 50.6 ± 4.5 (Figure 5(b)). Heat constitution and cold constitution rats caused a significant increase in the total number of ACFs observed as 71.2 ± 4.7 and 92.0 ± 3.7, respectively. The results showed that the number of ACF in the cold M group was the highest before the colorectal cancer lesion, followed by the heat M group. Combined with the results of pathological analysis, the results suggested that cold constitution and heat constitution will significantly increase the risk of precancerous lesions of colorectal cancer, especially the cold constitution.

### 4.6. The Changes of Colonic Collagen Morphology in Rats

**Figure 6:**
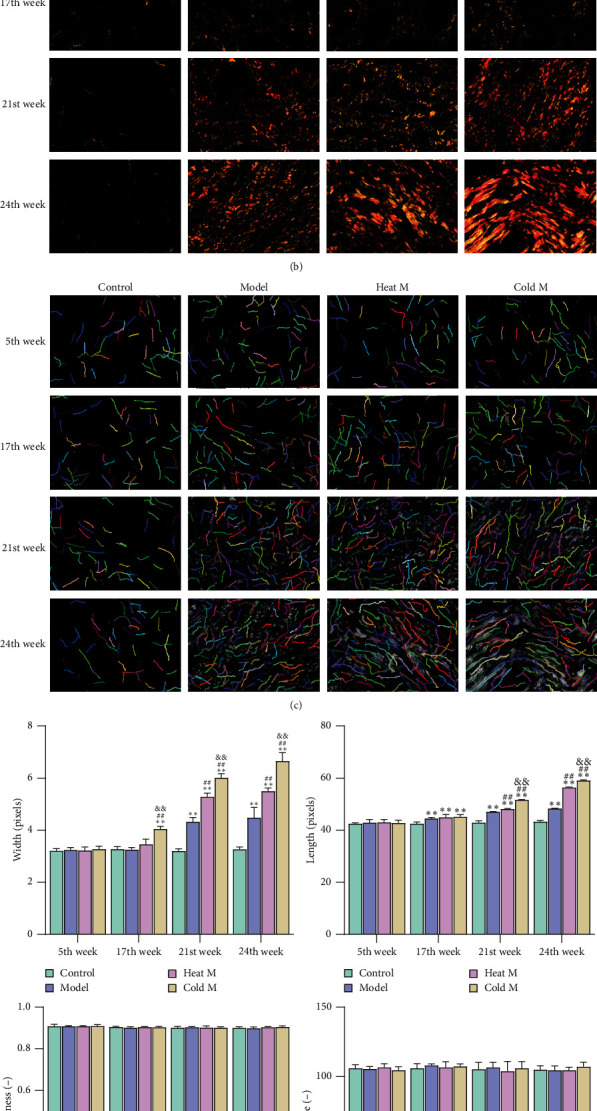
Changes in collagen morphology differ in different groups. (a). Representative images of collagen fiber arrangement characteristics (×400). B-C. The polarized light (b) and CT-FIRE (c) images of collagen were used to analyze the representative images (×400). (d). Quantitative analysis of the width, length, straightness, and angle of collagen. Compared with the control group: ^*∗∗*^*P* < 0.01, ^*∗*^*P* < 0.05; compared with the model group: ^##^*P* < 0.01, ^#^*P* < 0.05; compared with the heat M group: ^&&^*P* < 0.01, ^&^*P* < 0.05.

Collagen morphology, as one of the collagen characteristics, changed in the tumor development process (Figure 6(a)). The collagen fibers in normal colon tissue are thin, wavy, and dispersed, and we set it to grade I. The linearization of the collagen morphology in grade II is more obvious than that of grade I, and its density is higher, which is more common in precancerous lesions, such as atypical hyperplasia and tumor degeneration. The morphology in grades III-V was mainly found in CRC, and the collagenic linearization and density in grade III were more obvious than those in grade II. The morphology in grade IV was similar to that in grade III, but the density was higher and the collagen clumped into bundles. The collagen density in grade V increased significantly and was crosslinked into a plate. We found that at the 17th week, 18 cases were grade II (model: 6 cases; heat M group: 6 cases; cold M group: 6 cases). At the 21st week, 7 cases were grade II (model: 4 cases; heat M group: 2 cases; cold M group: 1 case), 8 cases were grade III (model: 2 cases; heat M group: 3 cases; cold M group: 3 cases), and 3 cases were grade IV (model: 0 cases; heat M group: 1 case; cold M group: 2 cases). At the 24th week, 7 cases were grade III (model: 4 cases; heat M group: 2 cases; cold M group: 1 case), 9 cases were grade IV (model: 2 cases; heat M group: 4 cases; cold M group: 3 cases), and 2 cases were grade V (model: 0 cases; heat M group: 0 cases; cold M group: 2 cases). Therefore, the morphology of colonic collagen in the cold M group showed a higher degree of collagen crosslinking than that in the model group and heat M group at the 21st and 24th weeks.

In order to quantitatively describe the morphological changes of collagen, we used the open-source software CT-FIRE to measure the morphological parameters of collagen fibers (including width, length, straightness, and angle, Figure 6(b)–6(d)). At the 5th week, there was no significant difference in the width, length, straightness, and angle of collagen between the control group, the model group, the heat M group, and the cold M group. At the 17th week (pathological hyperplasia of colon in model group, heat M group, and cold M group), the width and length of collagen in the model group, heat M group, and cold M group were higher than those in the control group (*P* < 0.01; Figure 6(d)). The collagenic width and length in the cold M group increased significantly, and there was no significant difference in stiffness and angle among the four groups. At the 21st and 24th week, the collagenic width and length in control group, model group, heat M group, and cold M group showed an increasing trend (*P* < 0.01; Figure 6(d)). The morphological observations indicated that collagen reorganization varied between cold and heat constitution from that in normal colon tissues, suggesting that the difference in collagen remodeling might be one of the reasons for the different colorectal carcinogenesis paradigm between the cold and heat constitution.

### 4.7. Quantitative Analysis of Colonic Collagen I/III and LOX/LOXL2 Expression and Distribution in Rats

#### 4.7.1. Colonic Collagen I/III Expression and Distribution in Rats

**Figure 7:**
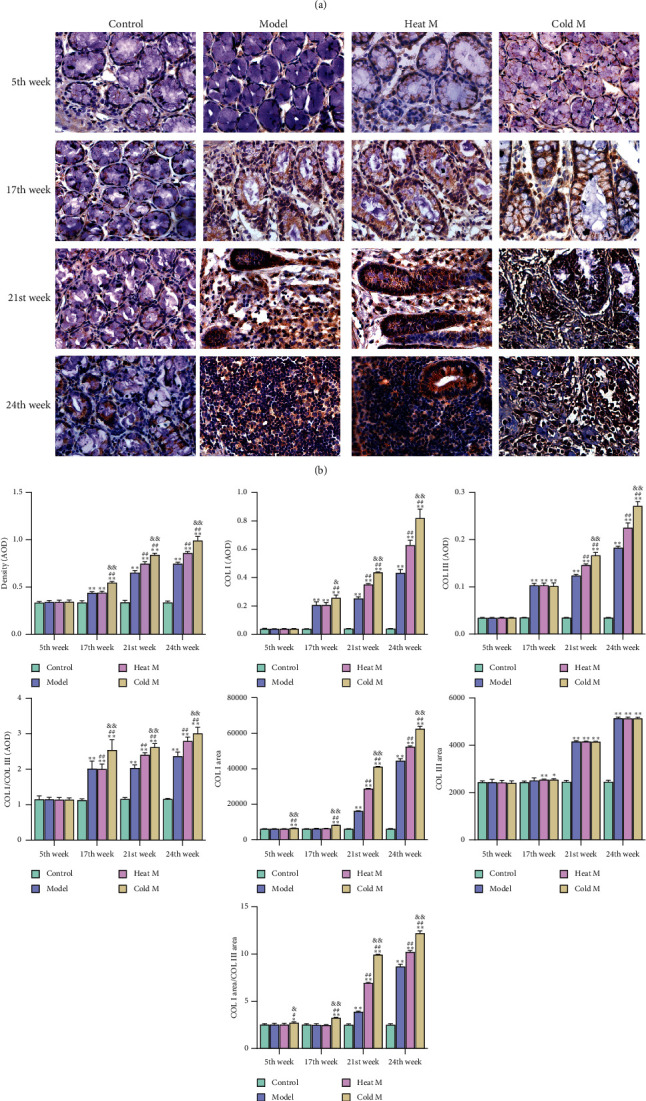
Comparison of collagen expression in different groups. Representative images of (a) COL I and (b) COL III in different groups (×400). (c). Differences in the expression of density in COL I COL III, COL I/COL III, COL I area, COL III area, and COL I area/COL III area. (d). The mRNA levels of COL I (COL1A1) and COL III (COL3A1). COL, collagen; AOD, average optical density. Compared with the control group: ^*∗∗*^*P* < 0.01, ^*∗*^*P* < 0.05; compared with the model group: ^##^*P* < 0.01, ^#^*P* < 0.05; compared with the heat M group: ^&&^*P* < 0.01, ^&^*P* < 0.05.

The content of total collagen detected by Sirius red staining in the colon of rats is shown in Figures 6(b) and 6(c). We found that in the 5th week, there was no significant difference in colon among the control group, model group, heat model group, and cold model group. At the 17th, 21st, and 24th weeks, the total collagen content of colon in the model group, cold M group, and heat M group was higher than that in the control group, while the content in the cold model group was significantly higher than that in model group and heat M group (*P* < 0.01; Figure 7(c)). This indicated that, from the 17th week, there was excessive collagen deposition in the colon of the model group, the cold M group, and the heat M group, while the colonic collagen deposition of the cold M group was the highest, and this trend continued until the end of the experiment.

The content, distribution area, and collagen ratio of COL I and III determined by immunohistochemistry and qRT-PCR are shown in Figure 7. In the 5th week, the COL I area and COL I area/COL III area in the cold M group were higher than those in the other three groups (*P* < 0.05; Figure 7(c)). In the 17th week, the expressions of COL I, COL III, and COL I/COL III in the cold M group, heat M group and model group were higher than those in the control group (*P* < 0.01), while the expressions of COL I, COL I/COL III, COLI area, and COL I area/COL III area in cold M group were higher than those in heat M group and model group (*P* < 0.05; Figure 7(c)). In the 21st and 24th weeks, the COL I area, COL III, COL I/COL III and COL I, COL III area, and COL I area/COL III area in the cold M group, heat M group, and model group were higher than those in the control group (*P* < 0.01; Figure 7(c)), and the COL I, COL III, COL I/COL III, COL I area, and COL I area/COL III area in the cold M group were higher than those in the heat M group and model group (*P* < 0.05; Figure 7(c)). Furthermore, the mRNA expressions of COL I (COL1A1) and COL III (COL3A1) in the cold M group, heat M group, and model group were higher than those in the control group at the 17th week (*P* < 0.05; Figure 7(d)). At the 21st and 24th weeks, the mRNA levels of COL I (COL1A1) and COL III (COL3A1) in the above four groups (control group, M group, heat M group, and cold M group) were ordered by increasing magnitude (*P* < 0.05; Figure 7(d)). This showed that the cold and heat constitution owned different degrees of collagen expression and distribution.

#### 4.7.2. The Level of LOX and LOXL2 in Colon Tissues of Rats

**Figure 8:**
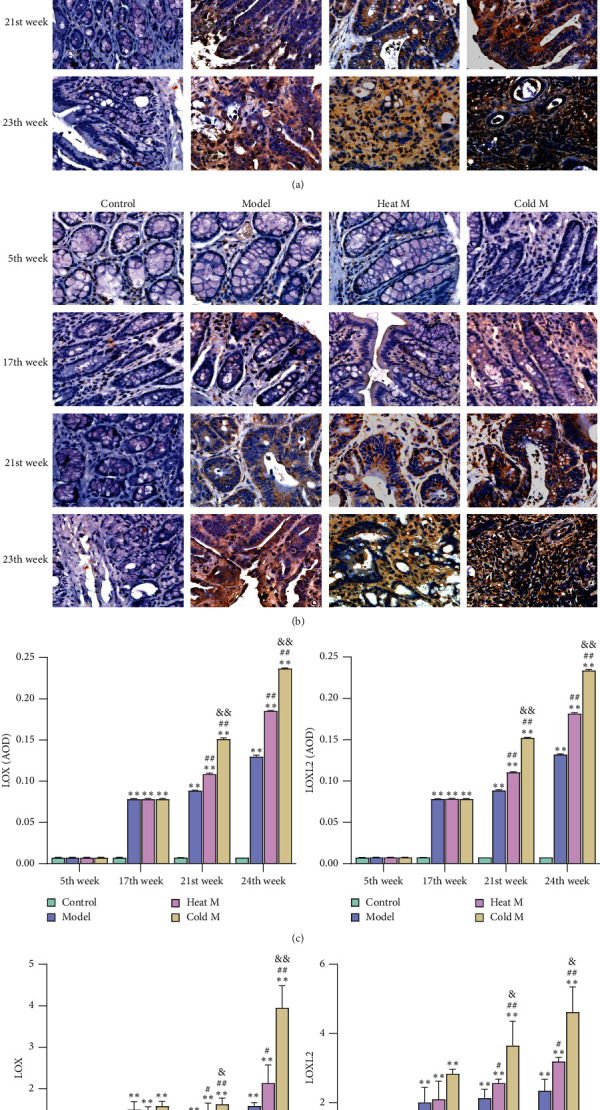
Comparison of expression levels with LOX and LOXL2 in different groups. Representative images of (a) LOX and (b) LOXL2 in different groups (×400). (c). Differences in the expression of LOX and LOXL2. (d). Differences in the mRNA levels of LOX (LOX) and LOXL2 (LOXL2). AOD, average optical density. Compared with the control group: ^*∗∗*^*P* < 0.01, ^*∗*^*P* < 0.05; compared with the model group: ^##^*P* < 0.01, ^#^*P* < 0.05; compared with the heat M group: ^&&^*P* < 0.01, ^&^*P* < 0.05.

The protein levels and gene expression of LOX and LOXL2 in the colon tissues were measured using qRT-PCR and immunostaining, respectively. The results (Figure 8) showed that, at the 5th week, there was no significant difference in LOX and LOXL2 among the control group, model group, heat model group, and cold model group. In the 17th week, the expression of LOX and LOXL2 in the colon of the model group, heat model group and cold model group had no significant difference, but they were all higher than those of the control group (*P* < 0.01; Figure 8(c)). In the 21st and 24th week, the expression of the control group, model group, heat M group, and cold M group showed an increasing trend in turn (*P* < 0.01; Figure 8(c)). Moreover, the results of the mRNA expression about LOX and LOXL2 were similar (*P* < 0.05; Figure 8(d)).

### 4.8. Baseline Characteristics in CRC Patients

According to the daily habits (drinking cold water at least 1500 mL per day or eating spicy food at least 5 days per week) and complexion, urine color, fecal appearance, and other clinical symptoms, 30 CRC patients were classified as having cold constitution and heat constitution. As shown in Table 5, a significant difference existed across the clinical stage and tumor metastasis between CRC patients with cold and heat constitution (*P* < 0.05), while there was no significant difference in sex, age, differentiation, and T stage (*P* > 0.05). The results were consistent with the results of animal experiments. Therefore, the tumor deterioration in CRC patients with cold and heat constitution is different.

### 4.9. Analysis of Colonic Collagen Characteristics in CRC Patients

**Figure 9:**
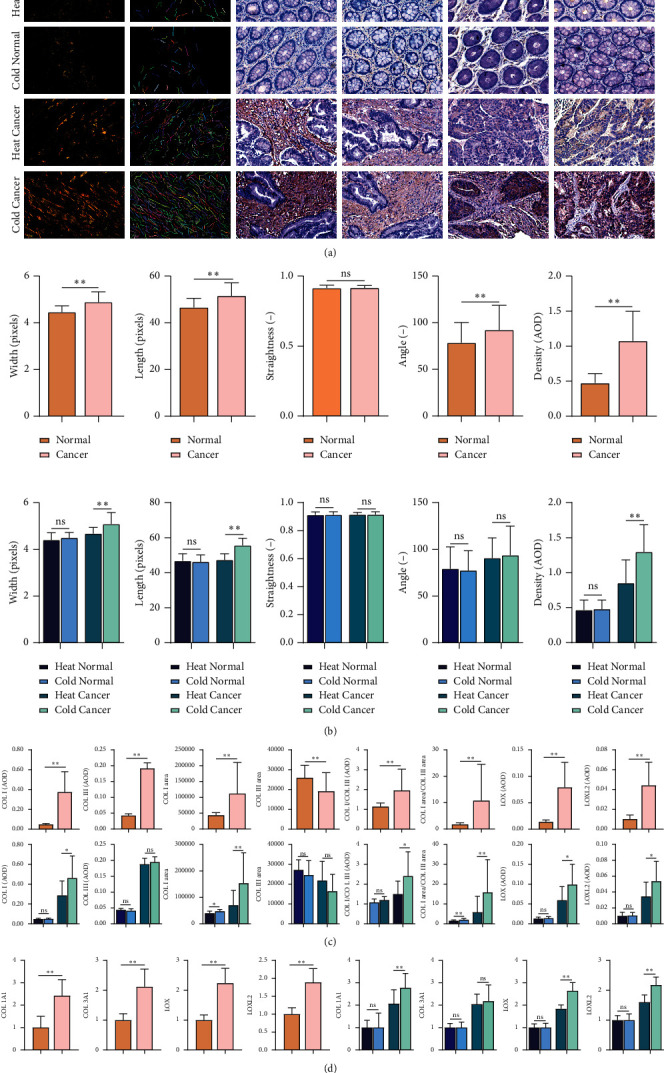
Comparison of collagen and collagenase in different tissue samples. (a) Representative images of polarized light and CT-FIRE, COL I COL III, LOX, and LOXL2 in different groups (×200). (b) Differences in the width, length, straightness, angle, and density of collagen. (c) Differences in the expression of COL I COL III, COL I/COL III, COL I area, COL III area and COL I area/COL III area, LOX, and LOXL2. (d) Differences in the mRNA levels of COL I (COL1A1), COL III (COL3A1), LOX (LOX), and LOXL2 (LOXL2). AOD, average optical density. ^*∗∗*^*P* < 0.01; ^*∗*^*P* < 0.05; ^*ns*^*P* > 0.05.

In the animal experiment, we found that the degree of collagen deposition was higher in the cold-constitution rats. In order to verify the clinical implications of this finding, we detected the collagen characteristics of colon tissues in clinical samples (Figure 9). First of all, the collagen-related parameters (length, width, and density) (Figure 9(b)), the expression and distribution area of COL I and III, and the expression of COL I/COL III, COL I area/COL III area, LOX and LOXL2 in cancer tissues were higher than those in normal tissues (Figure 9(c)). Secondly, there were significant differences in collagen width (*P* < 0.01), length (*P* < 0.01), and density (*P* < 0.01) of cancer tissues between cold-constitution and heat-constitution CRC patients, but there was no significant difference in normal tissues (Figure 9(b)). In addition, in cancer tissues, the expression and area of COL I, COL I/COL III, and COL I area/COL III area and the expression of LOX and LOXL2 in patients with cold constitution were significantly higher than those in patients with heat constitution. In normal tissues, COL I area and COL I area/COL III area in cold-constitution patients were also higher than those in heat-constitution ones (Figure 9(c)). The qRT-PCR results also showed that the mRNA expressions of COL I, COL III, LOX, and LOXL2 in tumor tissues were higher than those in normal tissues. Moreover, the mRNA expressions of COL I, LOX, and LOXL2 in the cancer tissues of cold-constitution patients were higher than those in heat-constitution patients (Figure 9(d)). The above results were consistent with the results of animal experiments. The abnormal changes of collagen characteristics in CRC patients with different constitutions were different, and the collagen deposition in cold-constitution CRC patients is more serious and earlier than that in heat-constitution CRC patients.

## 5. Discussion

Constitution is the basis of diagnosis, treatment, and prevention of diseases. If a disease is like a tree, then the body constitution is equivalent to the soil, and the occurrence and development patterns of the disease will be different in different types of constitution [6]. For constitution identification in CRC, the most important and unique constitutions are the cold and heat constitutions. In this study, ice water and capsaicin were used to induce rat models with basic symptoms of cold and heat constitution in order to simulate the cold constitution induced by long-term cold drink intake and heat constitution by long-term spicy food intake in human beings. The results of basic symptoms scores and energy metabolizing enzymes activities showed that the rat models with cold-constitution and heat-constitution were established successfully. Furthermore, the results showed that the number of ACF in the cold-constitution group is significantly higher than any other groups, followed by the heat-constitution group, and the results of the pathological evaluation are similar to the result of ACF screening, which suggests that the cold-constitution group has a higher risk of colonic precancerous lesions. On the other hand, at the end of the experiment, the incidence of tumors in both the heat- and cold-constitution group was 100% and higher than that in the model group; however, the number and size of tumors in the cold group were higher than those in other groups, followed by the heat-constitution group. In addition, lymph node metastasis was only found in the CRC of the cold-constitution group. In clinical cases, we also found that the tumor deterioration rate of patients with the cold constitution was higher than those with heat constitution, which was similar to that of animal experiments, which indicated the colorectal carcinogenesis paradigm is different between cold constitution and heat constitution. However, the potential molecular mechanisms underlying the cold or heat constitution affecting colorectal carcinogenesis paradigm remained unclear.

The microenvironment of tumor growth is likely to vary with each individual constitution, which may be one of the reasons that lead to different tumor growth depending on constitution [8]. ECM, as an important component of the microenvironment, was considered to be the trigger of cancer formation and metastasis [40, 41]. As we all know, ECM gradually showed the characteristics of fibrosis with excessive deposition and enlarged distribution of collagen during tumor formation [42, 43]. Hünerwadel et al. [44] have pointed out that ECM remodeling will affect the morphology and texture of the intestine, while the curled and stiff intestine is related to the severe fibrosis of ECM collagen. The collagens are often crosslinked and linearized, leading to increased stiffening of the tissue. Moreover, tissue fibrosis and stiffness caused by collagen deposition and crosslinking are signs of malignant progression [23]. COL I is the main structural protein in the interstitial ECM, which provides tensile strength and hardness for tissues [45]. The increased expression and deposition of COL I are positively correlated with the high metastasis rate of tumors [46, 47]. Studies have shown that the expression of COL I is more than any other subtypes of collagen in CRC [48, 49]. COL III is another rich subtype collagen of the ECM, near a COL I and its distribution; studies have shown that COL III can make cancer cells highly invasive with increased mobility [50]. Moreover, the ratio of COL I and COL III is considered to be one of the important marks of tumor progression [51]. On the other hand, collagen crosslinking accompanies tissue fibrosis, which increases the risk of malignancy [52, 53]. Lysyl oxidase (LOX) family, the copper-dependent amine oxidase, initiates the process of covalent intra- and intermolecular crosslinking of collagen by oxidatively deaminating specific lysine and hydroxylysine residues located in the telopeptide domains and is frequently elevated in tumors [54, 55]. Active LOX stiffens tissues and can compromise their function, and reduction of LOX activity tempers tissue stiffness and prevents fibrosis [56, 57].

As mentioned above, a number of studies have examined the roles of collagen deposition in cancer. It is known that the malignant progression of CRC is closely related to the colonic fibrosis induced by ECM collagen deposition [21]. In our study, we found the degree of colonic fibrosis in the cold constitution CRC group was significantly higher than that in other groups. Moreover, cold and heat constitution do affect the development of CRC, then the molecular mechanism related to collagen deposition may be the direct or indirect mechanism of cold or heat constitution affecting the CRC.

After analyzing the characteristics of ECM collagen between cold constitution and heat constitution in animal and clinical samples, we found that the density of collagen increased significantly, the arrangement of collagen was linear, and the width and length of collagen also increased in the cold-constitution CRC. Bayer et al. [58] have shown that ECM collagen deposition became more obvious with the increasing collagen density, length, width width, which could induce the growth of carcinoma. In this study, compared with other groups, the expression and distribution of COL I and III in cold constitution CRC were significantly increased, and COL I/COL III was also significantly increased, followed by the heat constitution group. With increasing collagen expression, distribution area, and collagen ratio, the degree of ECM deposition will be aggravated, which will activate tumor-associated signaling pathways and promote the occurrence and metastasis of CRC [21, 59]. Therefore, the ECM collagen of cold-constitution CRC had more obvious collagen deposition, promoting tumorous growth and migration, which may be one of the reasons for the higher metastatic ability in cold-constitution CRC. In addition, it is noteworthy that the distribution area of COLI in cold-constitution rats or patients at the precancerous stages was significantly increased, which suggested the ECM collagen characteristics changed in cold and heat constitution in the early stage of CRC. Previous studies have shown that overexpression of LOX and LOXL2 can increase the abnormal crosslinking of collagen, promote fibrosis remodeling, and accelerate the malignant progression of the tumor [60–62]. The results suggested that from the early stage of CRC, the expressions of LOX and LOXL2 of cold constitution CRC group increased and were higher than those of other groups, which resulted in the overabundance of crosslinking collagen deposition with more serious fibrosis in cold-constitution CRC group than in other groups. Generally speaking, CRC with constitution of cold and heat showed different degrees of collagen fibrosis in the early stage with different risk of precancerous lesions and malignant metastasis rates. Based on the above results, we speculate that cold and heat constitution may affect the colorectal carcinogenesis paradigm by influencing the early collagen deposition in colon tissue.

## 6. Conclusion

In summary, we attempted to examine an unspecific effect of heat constitution and cold constitution on the colorectal carcinogenesis paradigm. Our study suggested that the existence of cold and heat constitution may influence the colorectal carcinogenesis paradigm, which might be mediated by earlier collagen deposition. The established heat and cold constitution CRC models might be useful for clarifying mechanisms on the role of heat and cold constitution in colorectal cancer. This study might indicate that a patient's constitution should be considered at each stage of CRC treatment and provide an effective idea for the diagnosis and treatment of CRC patients with different constitution clinically.

## Figures and Tables

**Table 1:** Participant inclusion and exclusion criteria.

	The specific standard
Inclusion criteria	1. All the patients were pathologically diagnosed with colorectal cancer and without any presurgery treatment
	2. The patients had no history of smoking or drinking and no preceding history
	3. BMI was defined as normal (BMI = 18.5–24.9)
	4. Lifestyle habits were collected using self-report questionnaires: 1) cold constitution: habitually drinking cold water 4–6 cups (about 800–1200 mL) per day or prefer to eating cold food (e.g., ice-cream); 2) heat constitution: habitually eating spicy food at least 5 days per week
	5. The patients were evaluated for cold or heat signs by two senior clinicians. 1) Vital signs of cold constitutions: intolerance to cold, paler complexion, loose or watery stool, and clear urine; 2) main symptoms of heat constitution: tolerance to cold or intolerance to heat, dry mouth and thirst, redder complexion, dry and hard stool, and deep yellow urine
	6. All the patients have the capacity to understand the study and provide informed consent

Exclusion criteria	1. The patients had a family history of tumors
	2. Patients with dysfunction of liver and kidney, coronary heart disease, infectious diseases, and other autoimmune diseases were excluded

**Table 2:** Criteria for scoring the basic symptoms of cold and heat constitution models.

Indicators	0	1	2
Nose and lips	Pink	Pale white	Red
Toe claw	Pink	Pale white	Red
Tongue	Pink	Green and purple	Red
Feces	Normal shape	Soft rotten	Dry and hard
Temperament	Docile	Indifference	Irritable

**Table 3:** The number and incidence of colorectal tumors in each group at 21th and 24th weeks.

Time	Group	n	No. of tumor-bearing rats	Number of gross tumors (No. of rats)				The incidence of visible tumors in rats (%)
				0	1-2	3-4	>4	
21th week	Control	6	0	0	0	0	0	0.00
	Model	6	6	2	4	0	0	66.67
	Heat M	6	6	2	4	0	0	66.67
	Cold M	6	6	1	5	0	0	83.33

24th week	Control	6	0	0	0	0	0	0.00
	Model	6	4	2	4	0	0	66.67
	Heat M	6	6	0	6	0	0	100.00
	Cold M	6	6	0	5	1	0	100.00

**Table 4:** The number of rats with mucosal pathological tissue changes in different time periods.

						Adenocarcinoma		With lymphatic metastasis	
Week	Group	n	Normal	Atypical hyperplasia	Adenoma	Mucosal carcinoma	Infiltrating carcinoma	Metastasis	Tumor metastasis rate (%)
5	Control	6	6	0	0	0	0	0	0.00
	Model	6	6	0	0	0	0	0	0.00
	Heat M	6	6	0	0	0	0	0	0.00
	Cold M	6	6	0	0	0	0	0	0.00

17	Control	6	6	0	0	0	0	0	0.00
	Model	6	3	3	0	0	0	0	0.00
	Heat M	6	0	6	0	0	0	0	0.00
	Cold M	6	0	6	0	0	0	0	0.00

21	Control	6	6	0	0	0	0	0	0.00
	Model	6	0	0	4	2	0	0	0.00
	Heat M	6	0	0	2	3	1	0	0.00
	Cold M	6	0	0	1	2	3	0	0.00

24	Control	6	6	0	0	0	0	0	0.00
	Model	6	0	0	2	4	0	0	0.00
	Heat M	6	0	0	0	2	4	0	0.00
	Cold M	6	0	0	0	1	5	2	33.33

**Table 5:** Comparison of baseline characteristics in patients with cold constitution and heat constitution.

Baseline characteristics	Cold constitution (*n* = 15)	Heat constitution (*n* = 15)	*P*
*Gender*			
Male, *n* (%)	8 (53.33%)	6 (40%)	0.713
Female, *n* (%)	7 (46.67%)	9 (60%)	

*Age (years)*			
<50, *n* (%)	1 (6.67%)	3 (20%)	0.598
≥50, *n* (%)	14 (93.33%)	12 (80%)	

*Differentiation*			
Medium *n* (%)	13 (86.67%)	14 (93.33%)	1.000
Poor, *n* (%)	2 (13.33%)	1 (6.67%)	

*Clinical stage*			
Stages I-II, *n* (%)	3 (20%)	10 (66.67%)	0.025^*∗*^
Stages III-IV, *n* (%)	12 (80%)	5 (33.33%)	

*T stage*			
T1-T2, *n* (%)	5 (33.33%)	6 (40%)	0.705
T3-T4, *n* (%)	10 (66.67%)	9 (60%)	

*Tumor metastasis conditions*			
Distant metastasis	4 (26.67%)	1 (6.67%)	0.043^*∗*^
Lymphatic metastasis	8 (53.33%)	4 (26.67%)	
Nonmetastasis	3 (20%)	10 (66.67%)	

## Data Availability

The datasets supporting the conclusions of this article are included within the article.

## Abbreviations

CRC: Colorectal cancer
ECM: Extracellular matrix
ACF: Aberrant crypt foci
DMH: 1,2-Dimethylhydrazine hydrochloride
LOX: Lysyl oxidase
LOXL2: Lysyl oxide-like 2
COL: Collagen.
